# Supplementary material for: Identification of a TPP1 Q278X Mutation in an Iranian Patient with Neuronal Ceroid Lipofuscinosis 2: Literature Review and Mutations Update
Source: J Clin Med. 2022 Oct 29;11(21):6415. doi: 10.3390/jcm11216415 (PMC9653935; doi:10.3390/jcm11216415)
Supplement: Supplementary file 1 [file jcm-11-06415-s001.zip › jcm-1964336-supplementary.pdf]

Table S1: TPP1 variants identified in CLN2 patients.

Exon/Intron	cDNA variant	Protein posission	ACMG scoring	Zygosity	ACMG interpretation	References
Intron 1	c.18-3C>G	splice site	PM2,PP3	Hom	VUS	Kousi et al., 2012
Intron 1	c.17 + 1G>C	Consensus plice site position	?	Hom	P	Kousi et al., 2012
Exon 2	c.37dupC	p.L13fS	PVS1,PM2	Hom	LP	Kousi et al., 2012
Exon 2	c.38T>C	p.L13P	PM2,PP2	NA	VUS	https://www.ucl.ac.uk/ncl-disease/
Exon 2	c.79C>G	p.Q27E	PM2,PP2,BP4	NA	VUS	Ardicli et al., 2021
Intron 2	c.89+1G>A	Consensus plice site position	PVS1,PM2,PP5	Hom	P	Angural et al., 2021
Intron 02 - Exon 08	c.89+2_887del	p.?	NA	NA	NA	https://www.ucl.ac.uk/ncl-disease/
Intron 2	c.89 + 4A>G	Splicing effect	NA	NA	NA	Noher de Halac et al., 2005
Intron 2	c.89 + 5G>C	Splicing effect	NA	NA	NA	Kousi et al., 2012
Exon 3	c.139C>G	p.L47V	PM2,PP2,BP4	Hom	VUS	https://www.ucl.ac.uk/ncl-disease/
Exon 3	c.163C>T	p.Q55*	PVS1,PM2	NA	LP	https://www.ucl.ac.uk/ncl-disease/
Exon 3	c.177_180del	p.E59Dfs*20	PVS1,PM2	NA	LP	Chang et al., 2012
Exon 3	c.184T>A	p.S62T	PM2,PP2,PP5	Hom	VUS	Kousi et al., 2012
Exon 3	c.184_185del	p.S62Gfs*25	PVS1,PM2,PP5	NA	P	Lam et al., 2001

Exon 3	c.196C>T	p.Q66*	PVS1,PM2,PP5	NA	P	Sleat et al., 1999
Exon 3	c.228C>A	p.Tyr76*	PVS1,PM2	NA	LP	Johnson et al., 2020
Exon 3	c.229G>A	p.G77R	PS1,PM2,PP2,PP3,PP5	NA	P	Sleat et al., 1999
Exon 3	c.229G>T	p.G77*	PVS1,PM2	NA	LP	Chang et al, 2012
Intron 3	c.229+ 3G>C	Splicing effect	NA	NA	NA	https://www.ucl.ac.uk/ncl-disease/
Exon 4	c.237C>G	p.T79*	PVS1,PM2,PP5	Hom	P	Kousi et al., 2012
Exon 4	c.311T>A	p.L104*	PVS1,PM2,PP5	NA	P	Kohan et al., 2008
Exon 4	c.337dup	p.S113Ffs	PVS1,PM2	NA	LP	https://www.ucl.ac.uk/ncl-disease/
Exon 4	c.357dup	p.L120Sfs*18	PVS1,PM2,PP5	NA	P	Zhong et al., 2000
Exon 4	c.377_387del	Splicing effect	NA	NA	NA	Sleat et al., 1999
Exon 4	c.379C>T	p.R127*	PVS1,PM2,PP5	NA	P	Sleat et al., 1999
Exon 4	c.380G>A	p.R127Q	PM2,PP2,,PP3,PP5,	Hom	P	Zhong et al., 2000
Intron 4	c.380+ 55G>A	Splicing effect	NA	NA	NA	Mole et al., 2001
Intron 4	c.381–17_381–4del	Splicing effect	NA	NA	NA	Chang et al., 2012
Intron 4	c.381–2A>G	Splicing effect	NA	NA	NA	Zhong et al., 2000
Intron 4	c.381-1G>C	Splice defect	PVS1,PM2	Hom	P	Kousi et al., 2012

Exon 5	c.406_409dup	p.E139Gfs*1	PVS1,PM2	NA	LP	Chang et al., 2012
Exon 5	c.456G>C	p.R152S	PM2,PP2,,PP3,PP5,	Hom	LP	Sheth et al., 2018
Exon 5	c.457T>C	p.S153P	PM2,PP3,PP2	NA	VUS	Caillaud et al., 1999
Exon 5	c.471C>A	p.Y157*	PVS1,PM2,PP5	Hom	P	Kousi et al., 2012
Exon 5	c.481C>T	p.Q161*	PVS1,PM2,PP5	NA	P	https://www.ucl.ac.uk/ncl-disease/
Exon 5	c.497dupA	p.H166fs*	PVS1,PM2	NA	LP	Kousi et al., 2012
Intron 5	c.509-1G>C	Splice defect	PVS1,PM2,PP5	Hom	P	Sleat et al., 1999
Intron 5	c.509–1G>A	Splicing effect	NA	NA	P	Sleat et al., 1999
Exon 6	c.528del	p.P178Qfs*	PVS1,PM2	NA	LP	https://www.ucl.ac.uk/ncl-disease/
Exon 6	c.604C>A	p.P202T	PM2,PM5,PM1,PP2,PP3	NA	LP	Ardicli et al., 2021
Exon 6	c.605C>T	p.P202L	PM2,PM1,PP2,PP3	NA	LP	Mole et al., 2001
Exon06	c.616C>T	p.R206C	PM2,PM5,PM1,PP3,PP2,PP5	Hom	P	Berry-Kravis et al., 2000
Exon 6	c.617G>A	p.R206H	PM2,PM5,PM1,PP2,PP3,PP5	NA	LP	Kousi et al., 2012
Exon 6	c.617G>C	p.R206P	PP5,PM2,PM5,PP3,PM1,PP2	NA	P	https://www.ucl.ac.uk/ncl-disease/
Exon 6	c.622C>T	p.R208*	PVS1,PM2,PP5	Hom	P	Sleat et al., 1999
Exon 6	c.625T>C	p.Y209H	PM2,PM1,PP2,PP3, PP5	Hom	LP	Kousi et al., 2012

Exon 6	c.640C>T	p.Q214*	PVS1,PM2,PP5	Hom	P	Kousi et al., 2012
Exon 6	c.646G>A	p.V216M	PM2,PP2,PP3	NA	VUS	Wang et al., 2011
Exon 6	c.650G>T	p.G217D	PM2,PM5,PM1,PP2 ,PP5	NA	P	Chang et al., 2012
Exon 7	c.689delT	p.F230fs	PVS1,PM2,PP5	Com het	P	Angural., 2021
Exon 7	c.713C>G	p.S238*	PVS1,PM2	NA	LP	Kousi et al., 2012
Exon 7	c.729C>G	p.F243L	PM2,PP3,PP2	NA	VUS	https://www.ucl.ac.uk/ncl-disease/
Exon 7	c.731T>C	p.M244T	PM2,PP3,PP2	NA	LP	https://www.ucl.ac.uk/ncl-disease/
Exon 7	c.775del	p.R259Vfs*17	PVS1,PM2	NA	LP	Goldberg-Stern et al., 2009
Exon 7	c.790C>T	p.Q264*	PVS1,PM2	Hom	LP	Kousi et al., 2012
Exon 7	c.797G>A	p.R266Q	PM1,PP2,PM2	Hom	VUS	Kousi et al., 2012
Exon 7	c.802del	p.R268Gfs*8	PVS1,PM2	NA	LP	https://www.ucl.ac.uk/ncl-disease/
Exon 7	c.822_837del	p.L275*	PVS1,PM2	Hom	LP	Kousi et al., 2012
Exon 7	c.824T>C	p.L275P	PM1,PP2,PM2,PM5 ,PP3	NA	LP	Shen et al., 2013
Exon 7	c.827A>T	p.D276V	PM2,PP3,PP5	NA	P	Kohan et al. 2009
Exon 7	c.829G>A	p.V277M	PM1,PP2,PM2,PP3, PP5	NA	P	Ju et al., 2002
**Exon 7**	**c.832C>T**	**p.Q278***	**PVS1,PM2**	**Hom**	**LP**	**This study**

Exon 7	c.833A>C	p.Q278P	PM1,PP2,PM2,PM5,PP3	NA	LP	Ju et al., 2002
Exon 7	c.843G>T	p.M281I	PM1,PP2,PM2,PP3,	NA	LP	Kousi et al., 2012
Exon 7	c.851G>T	p.G284V	PM1,PP2, PM2,PP3,PP5	NA	P	Zhong et al., 2000
Exon 7	c.857A>G	p.N286S	PM1,PP2, PM2,PP5	Com het	P	Steinfeld et al., 2002
Exon 7	c.860T>A	p.I287N	PM1,PP2, PM2,PP3	NA	LP	Sleat et al., 1999
Intron 7	c.887–18A>G	Splicing effect	NA	NA	NA	Sleat et al., 1999
Intron 7	c.887-10A>G	varies	PM2,PP3,PP5	Hom	LP	Noher de Halac et al., 2005
Exon 8	c.887G>A	p.G296D	PM2,PP3,PP2,	NA	VUS	Reid et al., 2016
Exon 8	c.888_1066del	p.H298Lfs*	NA	NA	NA	Kousi et al., 2012
Exon 8	c.959T>G	p.V320G	PM2,PP3,PP2	NA	VUS	https://www.ucl.ac.uk/ncl-disease/
Exon 8	c.972_979del	p.S324Rfs	PVS1,PM2,PP5	NA	P	Sleat et al., 1999
Exon 8	c.984_986del	p.D328del	PM2,PM4,PM1	NA	VUS	Kousi et al., 2012
Exon 8	c.987_989delinsCTC	p.D329_D330delinsDS	PM2,PM1,PP2	NA	VUS	Kousi et al., 2012
Exon 8	c.1007A>G	p.Tyr336C	PM2,PP3,PM1,PP2	NA	VUS	https://www.ucl.ac.uk/ncl-disease/
Exon 8	c.1015C>T	p.R339W	PM2,PM5,PM1,PP2,PP3,PP5	NA	P	Kousi et al., 2012
Exon 8	c.1016G>A	p.R339Q	PM2,PM5,PM1,PP2,PP3,PP5	Hom	P	Kousi et al., 2012

Exon 8	c.1027G>A	p.E343L	PM2,PM5,PM1,PP2,PP3,PP5	NA	P	Sleat et al., 1999
Exon 8	c.1029G>C	p.E343D	PM2,PM5,PM1,PP2,PP3,PP5	NA	LP	Dy et al., 2015
Exon 8	c.1048C>T	p.R350W	PM2,PP2,PP3	NA	VUS	https://www.ucl.ac.uk/ncl-disease/
Exon 8	c.1049G>A	p.R350Q	PM2,PP3,PP2,	NA	VUS	https://www.ucl.ac.uk/ncl-disease/
Exon 8	c.1052G>T	p.G351V	PM2, PP3, PP2,	NA	VUS	https://www.ucl.ac.uk/ncl-disease/
Exon 8	c.1057A>C	p.T353P	PM2,PM5,PP2,PP3	NA	LP	Steinfeld et al., 2002
Exon 8	c.1058C>A	p.T353N	PM2,PM5,PP2,PP3, PP5	NA	LP	https://www.ucl.ac.uk/ncl-disease/
Exon 8	c.1062delG	p.L355Sfs*72	PVS1,PM2	Hom	LP	Kousi et al., 2012
Exon 8	c.1064 T>C	p.L355P	PM2,PP2,PP3	NA	VUS	Kousi et al., 2012
Intron 8	c.1075 + 2 T>G	splice defect	PVS1,PM2,PP5	Hom	P	Sleat et al., 1999
Intron 8	c.1076–2A>G	Splicing effect	NA	NA	NA	Caillaud et al., 1999
Intron 8	c.1076–2A>T	Splicing effect	NA	NA	NA	https://www.ucl.ac.uk/ncl-disease/
Exon 9	c.1093 T>C	p.C365R	PM2,PM5,PM1,PP2,PP3,PP5	NA	P	Sleat et al., 1999
Exon 9	c.1094G>A	p.C365Y	PM2,PM,PP2,PP3,PP5	NA	P	Sleat et al., 1999
Exon 9	c.1106dup	p.G370Wfs*33	PVS1,PM2	NA	LP	Itagaki et al. 2018
Exon 9	c.1107_1108del	p.G370Kfs*32	PVS1,PM2	NA	LP	Kohan et al., 2013

Exon 9	c.1145G> A	p.S382N	PVS1,PM2	NA	LP	Uygur et al. 2020
Intron 9	c.1145+2T>G	p.?	PVS1,PM2	NA	LP	https://www.ucl.ac.uk/ncl-disease/
Exon 10	c.1146C>G	p.S382R	PM2,PP2,PP3	NA	VUS	Kousi et al., 2012
Exon 10	c.1154 T>A	p.V385D	PM2,PP2,PP3,PP5	NA	LP	Sleat et al., 1999
Exon 10	c.1166G>A	p.G389E	PM2,PP2,PP3,PP5	NA	LP	Sleat et al., 1999
Exon 10	c.1204G>T	p.E402*	PVS1,PM2	NA	LP	Kousi et al., 2012
Exon 10	c.1226G>T	p.G409V	PM2,PM5,PM1,PP2,PP3,PVS1	NA	VUS	https://www.ucl.ac.uk/ncl-disease/
Exon 10	c.1226del	p.G409fs	PVS1,PM2	NA	LP	Lourenço et al., 2020
Exon 10	c.1239_1240ins6	p.S413_N414ins2	PM2,PM4	NA	VUS	https://www.ucl.ac.uk/ncl-disease/
Exon 10	c.1261 T>A	p.Y421N	PM2,PP2,PP3	NA	VUS	https://www.ucl.ac.uk/ncl-disease/
Exon 10	c.1266G>C	p.Q422H	PM2,PP2,PP3,PP5	NA	LP	Sleat et al., 1999
Intron 10	c.1266+1G>C	p.?	PVS1,PM2	NA	LP	https://www.ucl.ac.uk/ncl-disease/
Intron 10	c.1266 +5G>A	Splicing effect	NA	NA	NA	Sleat et al., 1999
Exon 11	C.1279del	p.V426V	PVS1,PM2	NA	LP	Noher de Halac et al., 2005
Exon 11	c.1284G>T	p.L428N	PM2,PM1,PP2	NA	VUS	Ju et al., 2002
Exon 11	c.1340G>A	p.R447H	PM2,PM5,PM1,PP2,PP3,PP5	NA	P	Sleat et al., 1999

Exon 11	c.1343C>T	p.A448V	PM2,PM5,PM1,PP2,PP3,PP5	Hom	LP	Kousi et al., 2012
Exon 11	c.1343C>A	p.A448D	PM2,PP3,PM5,PM1,PP2	NA	LP	Lourenco et al. 2020
Exon 11	c.1344del	p.Y449fs	PVS1,PM2	NA	LP	Lourenço et al., 2020
Exon 11	c.1351G>T	p.D451Y	PM2,PM1,PP2,PP3,	NA	VUS	https://www.ucl.ac.uk/ncl-disease/
Exon 11	c.1358C>A	p.A453D	PM2,PM1,PP2,PP3,	NA	VUS	Kohan et al., 2013
Exon 11	c.1358C>T	p.A453V	PM2,PM1,PP2,PP3,	NA	VUS	Kohan et al., 2009
Exon 11	c.1361C>A	p.A454E	PM2,PM1,P2,PP3,PP5	NA	LP	Sleat et al., 1999
Exon 11	c.1376A>C	p.Y459S	PM2,PM1,PP2,PP3,PP5	Hom	LP	Bhavsar et al., 2016
Exon 11	c.1379G>A	p.W460*	PVS1,PM2,PP5	NA	P	Zhong et al., 2000
Exon 11	c.1397 T>G	p.V466G	PM2,PP2,PP5	NA	P	Sun et al., 2013
Exon 11	c.1417G>A	p.G473R	PM2,PP3,PP2,PP5	NA	LP	Lam et al., 2001
Exon 11	c.1424C>T	p.S475L	PM2,PP3,PP2,PP5	NA	P	Sleat et al., 1999
Exon 11	c.1424del	p.S475Wfs*13	PVS1,PM2	NA	LP	Moore et al., 2008
Intron 11	c.1425 +1G>C	Splicing effect	NA	NA	NA	Kousi et al., 2012
Exon 12	c.1438G >A	p.V480M	PM2,PM5,PP3,PP2	Comp het	LP	Segura et al., 2021
Exon 12	c.1439 T>G	p.V480G	PM2,PP3,PP2,PP5	Hom	VUS	Elleder et al., 2008

Exon 12	c.1442 T>G	p.F481C	PM2,PP3,PP2	Hom	VUS	Ju et al., 2002
Exon 12	c.1444G>C	p.G482R	PM2,PP3,PP2	NA	VUS	Kousi et al., 2012
Exon 12	c.1467del	p.N489Lfs*29	PVS1,PM2	Hom	LP	https://www.ucl.ac.uk/ncl-disease/
Exon 12	c.1471del	p.H491Tfs*28	PVS1,PM2,PP5	NA	P	Dozières-Puyravel et al., 2019
Exon 12	c.1449dup	p.I484fs	PVS1,PM2,PP5	Het	P	Angural., 2021
Exon 12	c.1497delT	p.G501Afs*18	PVS1,PM2,PP5	Hom	P	Kousi et al., 2012
Exon 12	c.1501G>T	p.G501C	PM2,PP3,PP2,PP5	Hom	VUS	Kousi et al., 2012
Exon 12	c.1510A>T	p.N504Y	PM2,PP3,PP2,PP5	Hom	VUS	Kousi et al., 2012
Exon 12	c.1525C>T	p.Q509*	PVS1,PM2,PP5	NA	P	Caillaud et al., 1999
Exon 12	c.1547_1548del	p.F516fs*	PVS1,PM2,PP5	Hom	P	Kousi et al., 2012
Exon 12	c.1547_1548ins	p.D517Hfs*1	PVS1,PM2	NA	LP	Chang et al., 2012
Exon 12	c.1548_1551dup	p.F516*	PVS1,PP5,PM2	NA	P	Kousi et al., 2012
Intron 12	c.1551 + 1G>A	Splicing effect	NA	NA	NA	Wang et al., 2011
Intron 12	c.1551 + 1G>T	Splicing effect	PM2	NA	NA	Yu et al., 2015
Intron 12	c.1551 +5_1551 + 6dellinsTA	Splicing effect	NA	NA	NA	Kousi et al., 2012
Intron 12	c.1552–1G>C	Splicing effect	NA	NA	NA	Sleat et al., 1999

Exon 13	c.1552-1G > A	p.V518fs	PVS1,PM2	NA	LP	Lourenço et al., 2020
Exon 13	c.1593dup	p.E532Rfs*76	PVS1,PP5,PM2	NA	P	Dozières-Puyravel et al., 2019
Exon 13	c.1595dupA	p.Q534Pfs*74	PVS1,PM2	NA	LP	Sleat et al., 1999
Exon 13	c.1603G>C	p.G535R	PM2,PP3,PP2	NA	VUS	Kohan et al., 2013
Exon 13	c.1611_1621del	p.C537Wfs*67	PVS1,PM2,PP5	NA	P	Caillaud et al., 1999
Exon 13	c.1613C>A	p.S538Y	PM2,PP3,PP2	NA	VUS	Yu et al., 2015
Exon 13	c.1626G>A	p.W542*	PVS1,PM2,PP5	NA	P	https://www.ucl.ac.uk/ncl-disease/
Exon 13	c.1630C>T	p.P544S	PM2,PP3,PP2	NA	VUS	Zhong et al., 2000
Exon 13	c.1642T>C	p.W548R	PM2,PP3,PP2	NA	VUS	Zhong et al., 2000
Exon 13	c.1644G>A	p.W548*	PVS1,PM2	NA	LP	Kousi et al., 2012
Exon 13	c.1663del	p.A555Lfs*3	PVS1,PM2	NA	LP	https://www.ucl.ac.uk/ncl-disease/
Exon 13	c.1678_1679delCT	p.L560Tfs*47	PM2, PVS1,PP5	NA	LP	Sleat et al., 1999
